# Supplementary material for: Phenolic Compounds in Poorly Represented Mediterranean Plants in Istria: Health Impacts and Food Authentication
Source: Molecules. 2020 Aug 10;25(16):3645. doi: 10.3390/molecules25163645 (PMC7466117; doi:10.3390/molecules25163645)
Supplement: Supplementary file 1 [file molecules-25-03645-s001.pdf]

Table S1: Phenolic compound profile found in pomegranate.

Phenolic compounds	Group	Method	Origin of the samples	References
Epicatechin	flavonoid	HPLC-MS/MS	Slovenian and Croatian areas of Istria	29
Catechin	flavonoid	HPLC-MS/MS	Slovenian and Croatian areas of Istria	29
Gallic acid		HPLC-MS/MS; HPLC; HPLC-DAD-MS/MS	Slovenian and Croatian areas of Istria; from local markets, India; from the local market, Germany	29, 52, 60
Delphinidin-3,5-di-*O*-glucoside	Anthocyanin (*Flavonoids)*	HPLC-MS/MS	Slovenian and Croatian areas of Istria	29
Cyanidin-3,5-di-*O*-glucoside	Anthocyanin (*Flavonoids)*	HPLC-MS/MS	Slovenian and Croatian areas of Istria	29
Pelargonidin-3,5-di-*O*-glucoside	Anthocyanin (*Flavonoids)*	HPLC-MS/MS	Slovenian and Croatian areas of Istria	29
Cyanidin	Anthocyanin (*Flavonoids)*	HPLC -DAD	southern Turkey	105
Delphinidin	Anthocyanin (*Flavonoids)*	HPLC-DAD	southern Turkey	105
Pelargonidin	Anthocyanin (*Flavonoids)*	HPLC-DAD	southern Turkey	105
Punicalin	ellagitannin	HPLC-DAD; Sephadex LH-20 separation, MALDI-TOF; HPLC-MS; HPLC-DAD-MS/MS; HPLC-DAD-MS/MS	southern Turkey; from the local supermarket, WI, USA; Aligarh, India; Elche, Spain; from the local market, Germany	105. 56, 59, 48, 106
Pedunculagin	ellagitannin	HPLC-DAD; Sephadex LH-20 separation, MALDI-TOF; HPLC-MS; HPLC-DAD-MS/MS	southern Turkey; from the local supermarket, WI, USA; Aligarh, India; from the local market, Germany	105, 56, 59, 106
Punicalagin	ellagitannin	HPLC-DAD; Sephadex LH-20 separation, MALDI-TOF; HPLC-MS; HPLC-DAD-MS/MS; HPLC-DAD-MS/MS	southern Turkey; from the local supermarket, WI, USA; Aligarh, India; Elche, Spain; from the local market, Germany	105, 56, 59, 58, 106
Ellagic acid		HPLC-DAD; HPLC; HPLC-DAD-MS/MS	southern Turkey; from local markets, India; from the local market, Germany	105, 61, 106
Gallagic acid	ellagitannins	HPLC-MS	Aligarh, India	59
Ellagic acid esters of glucose	ellagitannins	HPLC-MS	Aligarh, India	59
Ellagitannins punicalagin	ellagitannins	HPLC-DAD-MS/MS	Elche, Spain	58
Ellagic acid hexoside	ellagitannins	HPLC-DAD-MS/MS	Elche, Spain	58

Tertgallic acid	Hydroxybenzoics	HPLC	from local markets, India	61
Delphinidin 3,5-diglucoside	Anthocyanin (*Flavonoids)*	HPLC-DAD-MS/MS	from the local market, Germany	106
Cyanidin 3,5-diglucoside	Anthocyanin (*Flavonoids)*	HPLC-DAD-MS/MS	from the local market, Germany	106
Pelargonidin 3,5-diglucoside	Anthocyanin (*Flavonoids)*	HPLC-DAD-MS/MS	from the local market, Germany	106
Delphinidin 3-glucoside	Anthocyanin (*Flavonoids)*	HPLC-DAD-MS/MS	from the local market, Germany	106
Cyanidin–pentoside–hexoside	Anthocyanin (*Flavonoids)*	HPLC-DAD-MS/MS	from the local market, Germany	106
Cyanidin 3-glucoside	Anthocyanin (*Flavonoids)*	HPLC-DAD-MS/MS	from the local market, Germany	106
Cyanidin 3-rutinoside	Anthocyanin (*Flavonoids)*	HPLC-DAD-MS/MS	from the local market, Germany	106
Pelargonidin 3-glucoside	Anthocyanin (*Flavonoids)*	HPLC-DAD-MS/MS	from the local market, Germany	106
Cyanidin-pentoside	Anthocyanin (*Flavonoids)*	HPLC-DAD-MS/MS	from the local market, Germany	106
Galloyl-hexose	Flavonoids	HPLC-DAD-MS/MS	from the local market, Germany	106
Digalloyl-hexose	Flavonoids	HPLC-DAD-MS/MS	from the local market, Germany	1106
HHDP-hexose	Ellagitannins	HPLC-DAD-MS/MS	from the local market, Germany	106
Ellagic acid-hexose	ellagitannis	HPLC-DAD-MS/MS	from the local market, Germany	106
Ellagic acid-pentose	ellagitannins	HPLC-DAD-MS/MS	from the local market, Germany	106
Ellagic acid-deoxyhexose	ellagitannins	HPLC-DAD-MS/MS	from the local market, Germany	106
Galloyl-HHDP-hexose	elagitannins	HPLC-DAD-MS/MS	from the local market, Germany	106
Casuarinin	ellagitannin	HPLC-DAD-MS/MS	from the local market, Germany	106
Ellagic acid derivative	ellagitanins	HPLC-DAD-MS/MS	from the local market, Germany	106
Ellagic acid dihexose	naturaelagitannins	HPLC-DAD-MS/MS	from the local market, Germany	106
Valoneic acid bilactone	Hydrolysable tannin	HPLC-DAD-MS/MS	from the local market, Germany	106
Lagerstannin B	Hydrolysable tannin	HPLC-DAD-MS/MS	from the local market, Germany	106
Lagerstannin B derivative	Hydrolysable tannin	HPLC-DAD-MS/MS	from the local market, Germany	106
Granatin B	ellagitannin	HPLC-DAD-MS/MS	from the local market, Germany	106
Castalagin derivative	ellagitannin	HPLC-DAD-MS/MS	from the local market, Germany	106
Lagerstannin C	ellagitannin	HPLC-DAD-MS/MS	from the local market, Germany	106
Punigluconin	ellagitannin	HPLC-DAD-MS/MS	from the local market, Germany	106
Brevifolin carboxylic acid	elagitannins	HPLC-DAD-MS/MS	from the local market, Germany	106
Protocatechuic acid	hydroxybenzoics	HPLC-DAD-MS/MS	from the local market, Germany	106
Protocatechuic acid-derivative	hydroxybenzoics	HPLC-DAD-MS/MS	from the local market, Germany	106

Vanillic acid-hexose	hydroxybenzoics	HPLC-DAD-MS/MS	from the local market, Germany	106
Caffeic acid hexose	hydroxybenzoics	HPLC-DAD-MS/MS	from the local market, Germany	106
Caffeic acid hexose derivative	hydroxybenzoics	HPLC-DAD-MS/MS	from the local market, Germany	106
Caffeic acid derivative	hydroxybenzoics	HPLC-DAD-MS/MS	from the local market, Germany	106
5-*O*-Caffeoylquinic acid	hydroxybenzoics	HPLC-DAD-MS/MS	from the local market, Germany	106
Ferulic acid-hexose	hydroxycinnamics	HPLC-DAD-MS/MS	from the local market, Germany	106
Coumaric acid	hydroxycinnamic acid	HPLC-DAD-MS/MS	from the local market, Germany	106
Dihydrokaempferol-hexose	*Flavonoids*	HPLC-DAD-MS/MS	from the local market, Germany	106

Table S2: Phenolic compound profile found in jujube.

**Phenolic compounds**	**Group**	**Method**	**Origin of the samples**	**Reference**
Protocatechuic acid	hydroxybenzoics	HPLC-UV VIS	Yulin, China	71
Cinnamic acid	hydroxycinnamics	HPLC-UV VIS	Yulin, China	71
Chlorogenic acid	hydroxycinnamics	HPLC-UV VIS; HPLC-DAD-MS/MS	Yulin, China; Korea	71, 75
Caffeic acid	hydroxybenzoics	HPLC-UV VIS; HPLC-DAD-MS/MS	Yulin, China; Korea	71; 75
Ferulic acid	hydroxycinnamics	HPLC-UV VIS	Yulin, China	71
Ellagic acid	natural phenol antioxidant	HPLC-UV VIS	Yulin, China	71
Catechin	flavonoid	HPLC-UV VIS; HPLC-MS/MS	Yulin, China; Slovenian and Croatian Istria	71, 30
Epicatechin	flavonoid	HPLC-UV VIS; HPLC-MS/MS; HPLC-DAD-MS/MS	Yulin, China; Slovenian and Croatian Istria; Korea	71, 30, 73
Rutin	flavonoids	HPLC-UV VIS	Yulin, China	71
Quercetin	flavonoids	HPLC-UV VIS	Yulin, China	71
Gallic acid	hydroxybenzoics	HPLC-UV VIS; HPLC-MS/MS	Yulin, China; Slovenian and Croatian Istria	71, 30
Delphinidin-3,5-di-*O*-glucoside	Anthocyanin (*Flavonoids)*	HPLC-MS/MS	Slovenian and Croatian Istria	30
Cyanidin-3,5-di-*O*-glucoside	Anthocyanin (*Flavonoids)*	HPLC-MS/MS	Slovenian and Croatian Istria	30
Pelargonidin-3,5-di-O-glucoside	Anthocyanin (*Flavonoids)*	HPLC-MS/MS	Slovenian and Croatian Istria	30
Procyanidin B2	proanthocyanidin	HPLC-DAD-MS/MS	Korea	73

Quercetin-3-*O*-robinobioside	flavonoids	HPLC-DAD-MS/MS; HPLC-DAD-MS/MS	Korea; Italy	73, 75
Quercetin-3-*O*-rutinoside	flavonoids	HPLC-DAD-MS/MS; HPLC-DAD-MS/MS	Korea; Italy	73, 75
Quercetin-3-*O*-galactoside	flavonoids	HPLC-DAD-MS/MS	Korea	73
Kaempferol-glucosyl-rhamnoside	flavonoids	HPLC-DAD-MS/MS	Korea	73
Quercetin-3-rutinoside	flavonoids	HPLC-DAD-MS/MS	Korea	75
(+/-)-Catechin	anthocyanin	HPLC-DAD-MS/MS	Korea	75
(-)-Catechin	anthocyanin	HPLC-DAD-MS/MS	Korea	75
Kaemferol-3-O-robinobioside	flavonoids	HPLC-DAD-MS/MS	Italy	75
Kaempferol-3-O-rutinoside	flavonoids	HPLC-DAD-MS/MS	Italy	75
Quercetin-3-*O*-R-L-arabnosyl-alfa-rhamnoside	flavonoids	HPLC-DAD-MS/MS	Italy	75
Quercetin-3-O-β-D-xylosyl-R-rhamnoside	flavonoids	HPLC-DAD-MS/MS	Italy	75

Table S3: Phenolic compound profile found in strawberry tree.

**Phenolic compounds**	**Group**	**Methods**	**Origin of the samples**	**Reference**
Gallic acids	hydroxybenzoics	GC-MS	Turkey	40
Protocatechuic acid	hydroxybenzoics	GC-MS	Turkey	40
Gentisic acid	hydroxybenzoics	GC-MS	Turkey	40
p-hydrobenzoic acid	hydroxybenzoics	GC-MS	Turkey	40
m-anisic acid	hydroxybenzoics	GC-MS	Turkey	40
Quercetin galloylhexoside derivatives	flavonoids	HPLC-MS/MS	Portugal	79
Quercetin-3-*O*-rutinoside	flavonoids	HPLC-MS/MS	Portugal	79
Quercetin-3-*O*-glucoside	flavonoids	HPLC-MS/MS	Portugal	79
Quercetin pentoside	flavonoids	HPLC-MS/MS	Portugal	79
Quercetin rhamnoside	flavonoids	HPLC-MS/MS	Portugal	79
Kaempferol hexoside	flavonoids	HPLC-MS/MS	Portugal	79

Myricetin rhamnoside	Flavonoids	HPLC-MS/MS; HPLC-MS/MS	Portugal; Portugal	79; 80
B-type proanthocyanidin trimers	*proanthocyanidins*	HPLC-MS/MS	Portugal	79
B-type proanthocyanidin tetramer	*proanthocyanidins*	HPLC-MS/MS	Portugal	79
B-type procyanidine dimer	*proanthocyanidins*	HPLC-MS/MS	Portugal	79
Galloylquinic acid	Gallotanni	HPLC-MS/MS; HPLC-MS/MS	Portugal; Portugal	79; 80
Galloylhexoside acid	Gallotanni	HPLC-MS/MS	Portugal	79
Galloylshiquimic acid	Gallotanni	HPLC-MS/MS	Portugal	79
(+)-Catechin	Flavonoid	HPLC-MS/MS	Portugal	79
Digalloylquinic acid	Gallotanni	HPLC-MS/MS; HPLC-MS/MS	Portugal; Portugal	79; 80
Digalloylquinic shikimic acid	Gallotanni	HPLC-MS/MS	Portugal	79
Strictinin elagitannin	flavan-3-ols and galloyil derivatives	HPLC-MS/MS	Portugal	79
Delphinidin-3-*O*-glucoside	anthocyanin (*flavonoids)*	HPLC-MS/MS	Portugal	79
Cyanidine-3-*O*-glucoside	anthocyanin (*flavonoids)*	HPLC-MS/MS	Portugal	79
Cyanidine-3-*O*-pentoside	anthocyanin (*flavonoids)*	HPLC-MS/MS	Portugal	79
Gallic acid glucoside	hydroxybenzoics	HPLC-MS/MS	Portugal	80
Quinic acid derivative		HPLC-MS/MS	Portugal	80
Proanthocyanidin dimer	anthocyanin (*flavonoids)*	HPLC-MS/MS	Portugal	80
Galloylshikimic acid	Gallotanni	HPLC-MS/MS	Portugal	80
Digalloylshikimic acid	gallotanni	HPLC-MS/MS	Portugal	80
Catechin monomer	flavonoids	HPLC-MS/MS	Portugal	80
Proanthicyanidine trimer	anthocyanin (*flavonoids)*	HPLC-MS/MS	Portugal	80
Strictinin ellagitannin	Ellagitannin	HPLC-MS/MS	Portugal	80
Ellagitannin derivative	Ellagitannin	HPLC-MS/MS	Portugal	80
Galloyl derivative	Gallotanni	HPLC-MS/MS	Portugal	80
Trigalloylshikimic acid	Gallotanni	HPLC-MS/MS	Portugal	80
Quercetin glucoside	Flavonoids	HPLC-MS/MS	Portugal	80
Gallotannin	Gallotanni	HPLC-MS/MS	Portugal	80

Ellagic acid rhamnoside	Ellagitannin	HPLC-MS/MS	Portugal	80
Delphinidin-3-galactoside	Anthocyanin (*Flavonoids)*	HPLC-DAD	Salamanca (West Spain)	82
Cyanidin-3-galactoside	Anthocyanin (*Flavonoids)*	HPLC-DAD	Salamanca (West Spain)	82
Cyanidin-3-glucoside	Anthocyanin (*Flavonoids)*	HPLC-DAD	Salamanca (West Spain)	82
Cyanidin-3-arabinoside	Anthocyanin (*Flavonoids)*	HPLC-DAD	Salamanca (West Spain)	82
Myricetin-3-xyloside	flavonoids	HPLC-DAD	Salamanca (West Spain)	82
Quercetin-3-xyloside	flavonoids	HPLC-DAD	Salamanca (West Spain)	82
Gallocatechin	flavonoids	HPLC-DAD	Salamanca (West Spain)	82
Gallocatechin-4,8-catechin	flavonoids	HPLC-DAD	Salamanca (West Spain)	82
Epicatechin-4,8-catechin	flavonoids	HPLC-DAD	Salamanca (West Spain)	82
Epicatechin-4,6-catechin	flavonoids	HPLC-DAD	Salamanca (West Spain)	82
Epicatechin-4,8-epicatechin	flavonoids	HPLC-DAD	Salamanca (West Spain)	82
Epicatechin-4,6-catechin dimers	flavonoids	HPLC-DAD	Salamanca (West Spain)	82
Epicatechin	flavonoids	HPLC-DAD	Salamanca (West Spain)	82
Epicatechin-4-8-epicatechin-4,8-catechin	flavonoids	HPLC-DAD	Salamanca (West Spain)	82
Epicatechin-4,8-epicatechin-4,8-epicatechin	flavonoids	HPLC-DAD	Salamanca (West Spain)	82
Delphinidin-3-*O*-galactoside	Anthocyanin (*Flavonoids)*	HPLC-DAD	Pisa in Italy	12
Cyanidin-3-*O*-glucoside	Anthocyanin (*Flavonoids)*	HPLC-DAD	Pisa in Italy	12
Cyanidin-3-*O*-arabinoside	Anthocyanin (*Flavonoids)*	HPLC-DAD	Pisa in Italy	12
4-Arbutin	glycoside	HPLC-DAD	Pisa in Italy	12
ß-D-Glucogalline	glucogalline	HPLC-DAD	Pisa in Italy	12
3-*O*-Galloylquinic acid	gallotanni	HPLC-DAD	Pisa in Italy	12
Gallic acid-4-*O*-ß-D-glucopyranoside	hydroxybenzoics	HPLC-DAD	Pisa in Italy	12
5-*O*-Galoylquinic acid	gallotanni	HPLC-DAD	Pisa in Italy	12
5-*O*-Galloylshikimic acid	gallotanni	HPLC-DAD	Pisa in Italy	12
3-*O*-Galloylshikimic acid	gallotanni	HPLC-DAD	Pisa in Italy	12

Table S4: Phenolic compound profile found in hackberry tree.

Phenolic compounds	Group	Methods	Origin of the samples	References
Acacetin 7-O-glucoside	flavonoids	HPLC-MS / NMR; HPLC-DAD	Italy; Italy	31, 33
Isovitexin	flavonoids	HPLC-MS / NMR; HPLC-DAD	Italy; Italy	31, 33
Cytisoside	flavonoids	HPLC-MS / NMR; HPLC-DAD	Italy; Italy	31, 33
Caffeic acid derivatives	hydroxybenzoics	HPLC-DAD	Italy	33
Chlorogenic acid	hydroxycinnamics	HPLC-DAD	Italy	33
2"-*O*-a-L-Rhamnopyranosylvitexin	flavonoids	HPLC-DAD	Italy	33
Vitexin	flavonoids	HPLC-DAD	Italy	33
2"-O-a-L-rhamnopyranosyl-7-O-methylvitexin	flavonoids	HPLC-DAD	Italy	33
Epicatechin	flavonoids	HPLC–MS/MS	Slovenian and Croatian Istria	42
Gallic acid	hydroxybenzoics	HPLC–MS/MS	Slovenian and Croatian Istria	42
Vanillic acid	hydroxybenzoics	HPLC–MS/MS	Slovenian and Croatian Istria	42
3,5-Dihydroxybenzaldehyde		HPLC–MS/MS	Slovenian and Croatian Istria	42
Delphinidin-3,5-di-*O*-glucoside	Anthocyanin (*Flavonoids)*	HPLC–MS/MS	Slovenian and Croatian Istria	42
Cyanidin-3,5-di-*O*-glucoside	Anthocyanin (*Flavonoids)*	HPLC–MS/MS	Slovenian and Croatian Istria	42
Pelargonidin-3,5-di-*O*-glucoside	Anthocyanin (*Flavonoids)*	HPLC–MS/MS	Slovenian and Croatian Istria	42

Table S5: Phenolic compound profile found in fig.

Phenolic compounds	Group	Methods	Origin of the samples	References
Cyanidin as aglycone	anthocyanin	HPLC-DAD -MS/MS	Salamanca (Spain)	89
Pelargonidine derivates	anthocyanin	HPLC-DAD -MS/MS	Salamanca (Spain)	89
Peonidin-3-rutinoside	Anthocyanin (*Flavonoids)*	HPLC-DAD -MS/MS	Salamanca (Spain)	89
5-Carboxypyranocyanidin-3-rutinoside	Anthocyanin (*Flavonoids)*	HPLC-DAD -MS/MS	Salamanca (Spain)	89
Cyanidin 3-rutinose dimer	Anthocyanin (*Flavonoids)*	HPLC-DAD -MS/MS	Salamanca (Spain)	89
Catechin	*Flavonoids*	HPLC-DAD -MS/MS	Salamanca (Spain)	89
Epicatechin residues	*Flavonoids*	HPLC-DAD -MS/MS	Salamanca (Spain)	89

(Epi)catechin-(4-8)-cyanidin-3-glucoside	*Flavonoids*	HPLC-DAD -MS/MS	Salamanca (Spain)	89
(Epi)catechin-(4-8)-cyanidin-3-rutinoside	*Flavonoids*	HPLC-DAD -MS/MS	Salamanca (Spain)	89
Cyanidin-3,5-diglucoside	Anthocyanin (*Flavonoids)*	HPLC-DAD -MS/MS	Salamanca (Spain)	89
(Epi)catechin-(4-8)-pelargonidine-3-rutinoside	*Flavonoids*	HPLC-DAD -MS/MS	Salamanca (Spain)	89
Cyanidin-3-malonylglicosyl-5-glucoside	Anthocyanin (*Flavonoids)*	HPLC-DAD -MS/MS	Salamanca (Spain)	89
Cyanidin-3-glucoside	Anthocyanin (*Flavonoids)*	HPLC-DAD -MS/MS	Salamanca (Spain)	89
Cyanidin-3-rutinoside	Anthocyanin (*Flavonoids)*	HPLC-DAD -MS/MS	Salamanca (Spain)	89
Pelargonidine-3-glucoside	Anthocyanin (*Flavonoids)*	HPLC-DAD -MS/MS	Salamanca (Spain)	89
Pelargonidine-3-rutinoside	Anthocyanin (*Flavonoids)*	HPLC-DAD -MS/MS	Salamanca (Spain)	89
				89
Cyanidin-3-malonylglucoside	Anthocyanin (*Flavonoids)*	HPLC-DAD -MS/MS	Salamanca (Spain)	89
Luteolin 6Chexose-8Cpentose	*Flavonoids*	HPLC-DAD -MS/MS	Spain different varieties	107
Apigeninrutinoside	*Flavonoids*	HPLC-DAD -MS/MS	Spain different varieties	107
Kaempferolrutinoside	*Flavonoids*	HPLC-DAD -MS/MS	Spain different varieties	107
Quercetineacetilglucoside	*Flavonoids*	HPLC-DAD -MS/MS	Spain different varieties	107
Quercetinrutinoside	*Flavonoids*	HPLC-DAD -MS/MS	Spain different varieties	107
Quercetineglucoside	*Flavonoids*	HPLC-DAD -MS/MS	Spain different varieties	107
Cyanidin-3-glucoside	Anthocyanin (*Flavonoids)*	HPLC-DAD -MS/MS	Spain different varieties	107
Cyanidin-3-rutinoside	Anthocyanin (*Flavonoids)*	HPLC-DAD -MS/MS	Spain different varieties	107

Table S6: Phenolic compound profile found in globe artichoke.

**Phenolic compounds**	**Group**	**Methods**	**Origin of the samples**	**Reference**
1-caffeoylquinic acid	phenolic compound	HPLC-MS/MS	Italy	93
3-caffeoylquinic acid	phenolic compound	HPLC-MS/MS	Italy	93
5-caffeoylquinic acid	phenolic compound	HPLC-MS/MS	Italy	93
Luteolin-7-O-rutinoside	*Flavonoids*	HPLC-MS/MS	Italy	93
Luteolin-7-O-glucoside	*Flavonoids*	HPLC-MS/MS	Italy	93
Luteolin-7-O-glucuronide	*Flavonoids*	HPLC-MS/MS	Italy	93

3-5-Dicaffeoylquinic acid	phenolic compound	HPLC-MS/MS	Italy	93
Apigenin-7-O-rutinoside	*Flavonoids*	HPLC-MS/MS	Italy	93
1,5-Dicaffeoylquinic acid	phenolic compound	HPLC-MS/MS	Italy	93
Apigenin-7-O-glucoside	*Flavonoids*	HPLC-MS/MS	Italy	93
Apigenin-7-O-glucuronide	*Flavonoids*	HPLC-MS/MS	Italy	93
Apigenin malonyl-glucoside	*Flavonoids*	HPLC-MS/MS	Italy	93
Luteolin	*Flavonoids*	HPLC-MS/MS	Italy	93
Apigenin	*Flavonoids*	HPLC-MS/MS	Italy	93
Hesperetin	Flavanone (*Flavonoids)*	HPLC-MS/MS	Italy	93
Chlorogenic acid	hydroxycinnamics	HPLC-DAD	Germany	108
Cynaroside	*Flavonoids*	HPLC-DAD	Germany	108
Syringic acid	hydroxybenzoics	HPLC-DAD	Tunisia	109
Myricitrin	*Flavonoids*	HPLC-DAD	Tunisia	109
p-Coumaric acid	hydroxycinnamics	HPLC-DAD	Tunisia	109
Quercetin	*Flavonoids*	HPLC-DAD	Tunisia	109
Naringenin	Naringenin	HPLC-DAD	Tunisia	109
